# Passing rail traffic reduces bat activity

**DOI:** 10.1038/s41598-021-00101-3

**Published:** 2021-10-19

**Authors:** Paul Jerem, Fiona Mathews

**Affiliations:** 1 Faculty of Science, Animal Ecology, Vrije Universiteit Amsterdam, Amsterdam, The Netherlands; 2 Department of Evolution, Behaviour and Environment, School of Life Sciences, University of Sussex, Falmer, UK

**Keywords:** Conservation biology, Urban ecology

## Abstract

Rail transport is expanding, with a global increase in infrastructure of up to one-third predicted by 2050. Greater reliance on rail is expected to benefit the environment at a planetary level, by mitigating transport-related carbon emissions**.** However, smaller-scale, more direct consequences for wildlife are unclear, as unlike roads, railway impacts on animal ecology are rarely studied. As a group, bats frequently interact with transport networks due to their broad distribution and landscape-scale movements. Additionally, their nocturnality, and use of echolocation mean bats are likely to be affected by light and noise emitted by trains. To investigate whether passing trains affect bat activity levels, we monitored the two most abundant UK species using ultrasonic detectors at 12 wooded rail-side sites in southern England. Activity fell by ≥ 30–50% each time a train passed, for at least two minutes. Consequently, activity was reduced for no less than one-fifth of the time at sites with median rail traffic, and two-thirds or more of the time at the busiest site. Such activity changes imply repeated evasive action and/or exclusion from otherwise favourable environments, with potential for corresponding opportunity or energetic costs. Hence, disturbance by passing trains may disadvantage bats in most rail-side habitats.

## Introduction

Rail transport networks are expanding, with a global increase in infrastructure of up to one-third predicted by 2050^1^. Moving people and goods by train produces fewer greenhouse gas emissions per unit kilometre than either road or domestic air transport^2^. Consequently, increased reliance on rail is expected to benefit the environment at a planetary level, by reducing the contribution of transport to climate change^3^. However, smaller-scale, more direct consequences for wildlife are less well understood, as unlike roads, the impacts of railways on animal ecology have rarely been studied^4^.

While comprising only a fraction of the length of the world’s roads^5^, the worldwide rail network is extensive, incorporating approximately 1.1 million kilometres of track^6^. As such, rail transport’s capacity for direct ecological impact is considerable. Railways share a number of characteristics with roads—both are linear structures associated with light, noise and chemical pollution^7,8^. And, both can act as barriers^9,10^ and sources of mortality for wildlife^11–14^. Nonetheless, key differences exist which limit application of the substantial body of road ecology research^15,16^ to rail contexts. For example, traffic flow is much lower on railways^14,17^, and trains are larger, and often operate at greater speeds than road vehicles, producing considerably higher intensity broad band noise and vibration^18–21^. Also, railways tend to have more extensive margins^22^ and experience fewer vehicle emitted chemical pollutants^23^. Together, these distinctions confirm a clear need for specific rail ecology research.

To date, most investigations into the effects of railways on wildlife have focused on wildlife-train collisions as a source of mortality^23,24^. Yet, non-lethal disturbance effects are also likely to be important for sensitive species. Bats are one such potentially susceptible group. Their exceptionally broad distribution (across all regions except the Arctic and Antarctic, and some remote islands^25^) and ability to fly long distances, inevitably leads to frequent contact with human transport infrastructure. Moreover, many species use linear landscape features and edge habitats for commuting and foraging^26–29^, making leafy railway verges attractive environments^22^. But, nocturnality and a general dependence on sound as a primary sensory modality mean bats appear more likely than most taxa to be affected by the light and noise emitted by trains. Visual observations suggest bats approaching roads take evasive action, reversing course when vehicles approach^30^, or when traffic noise reaches a threshold level^31^. As trains present a substantially larger, and often faster stimulus than road vehicles, their passing might be expected to evoke earlier and/or more long-lasting responses. Likewise, the distinct frequency and temporal structure of rail noise may lead to further contrasting repercussions. Train noise usually contains more energy at higher frequencies than road noise. This results from aerodynamic, traction and fan noise, increasing proportionally with speed^21^, and squealing/flanging due to track defects or curvature^32^, which can generate high intensity ultrasonic frequencies^33^. Additionally, intervals between trains are typically longer than among vehicles on busy stretches of road, resulting in more intermittent noise along railways. Both factors have been found to influence foraging efficiency in bats. But, where high bandwidth noise appears more problematic than traffic noise^34,35^, intermittent sources may be easier to compensate for than those emitting continuous noise^36^. Regardless, either mechanism—repeated evasive action, or impeded foraging—could incur deleterious energetic and/or opportunity costs. Particularly along busier lines where trains might pass every few minutes, and possibly even to the extent where rail-side verges could act as ecological traps^37^.

Common and soprano pipistrelle bats (*Pipistrellus pipistrellus*, and *Pipistrellus pygmaeus* respectively) are frequently sympatric^38^, morphologically similar insectivorous species^39^, which are abundant in Europe and share broadly comparable aerial hawking foraging habits^40^. Both frequently use edge habitats and linear landscape features, including those associated with transport infrastructure^28,41–44^, with evidence suggesting common pipistrelles in particular favour rail-side habitats^22^. In wooded habitat, both species show little response to artificial night lighting^45,46^. But, while the hearing range of *P. pygmaeus* is not well understood, a sizeable overlap exists between *P. pipistrellus* sensitivity^47^ and noise emitted by trains. Hence, these species represent a valuable opportunity to improve our understanding of direct ecological effects of railways on bats.

To investigate whether passing trains might affect *P. pipistrellus* and *P. pygmaeus* (henceforth ‘pipistrelle’) activity levels, we monitored their ultrasonic calls at 12 wooded rail-side sites in southern England. We tested if variation in numbers of bat passes was associated with the timing of train passes, the intensity and frequency of noise emitted, train length, train speed, or train density. We predicted passing trains would induce evasive action, reducing pipistrelle activity around each train pass, with the duration of reduced activity dependent on stimulus magnitude.

## Results

**Figure 1:**
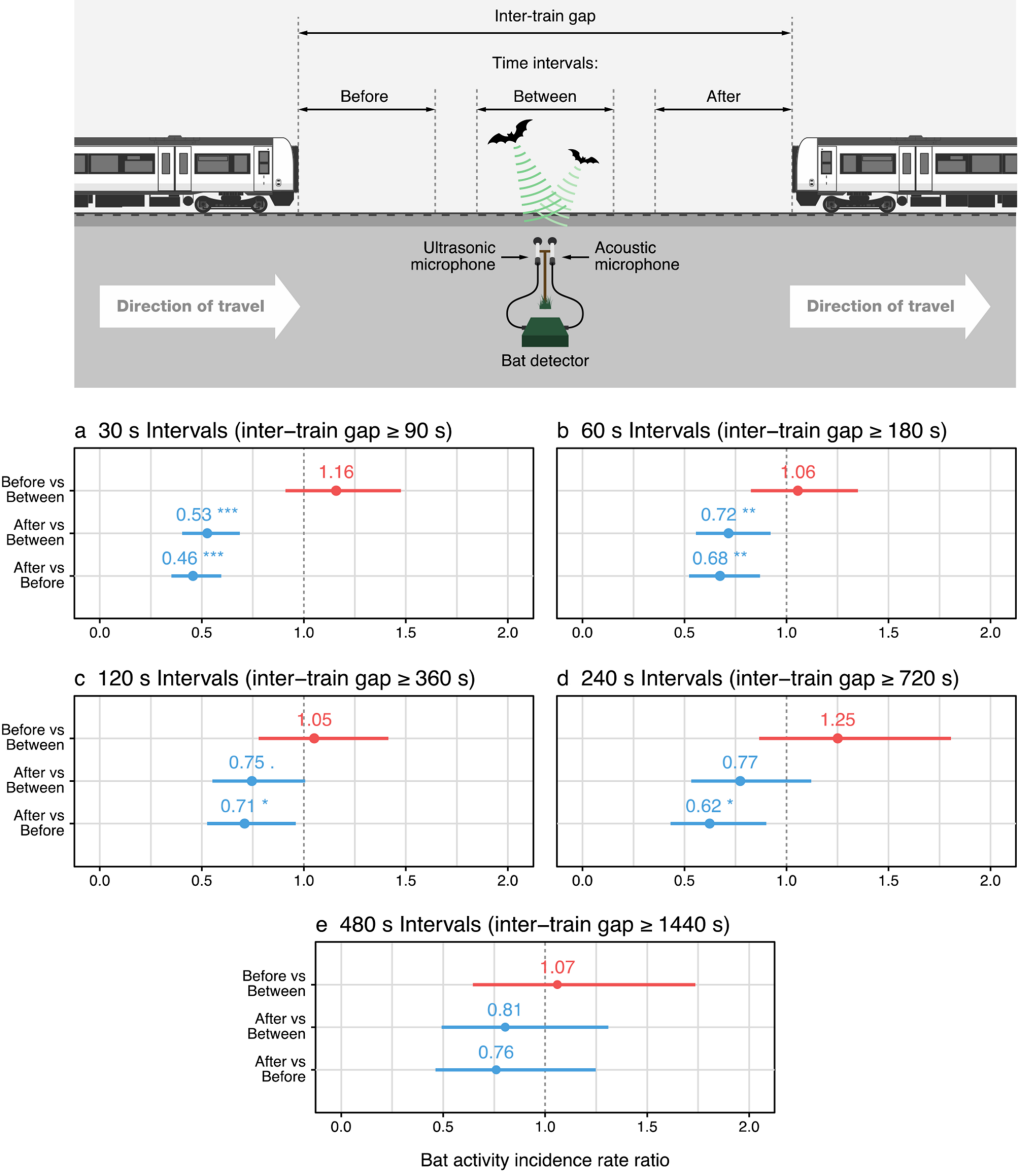
Schematic of the data collection setup, and incidence rate ratios (± 95% confidence intervals) comparing bat activity from before, after and between train passes in intervals of (**a**) 30 s, (**b**) 60 s, (**c**) 120 s, (**d**) 240 s and (**e**) 480 s duration. Each plot row provides the incidence rate ratio of the first interval type (data-point and confidence interval) against the second (vertical dotted line) noted on the y-axis. Asterisks indicate the significance level of a given comparison, (no asterisk—not significantly different; . p ≤ 0.1 *p ≤ 0.05; **p ≤ 0.01; ***p ≤ 0.001).

We recorded bat activity during 856 inter-train gaps (see Fig. 1. and Methods for definition) ≥ 90 s in length (mean(SD) duration = 490.5 (624.0) s, min = 90.2 s, max = 9984.4 s), with the number of train passes varying between sites from 1.7 (0.8) to 19.3 (1.5) per hour (mean(SD) across all sites = 8.6 (6.1) trains per hour, Supplementary Table 1). Maximum SPL during train passes was 107.3 (5.7) dB (min = 87.8 dB, max = 125.4 dB), while train speed varied from 2.5–166.5 km hr^−1^ (mean (SD) = 100. 9(32.7) km hr^−1^), and train length from 18.5–252.0 m (mean (SD) = 161. 9(65.4) m). Of the 6,255 pipistrelle passes recorded, 5,798 were made by common pipistrelles, and 457 were made by soprano pipistrelles (nightly total of bat passes recorded during inter-train gaps per 3.5 h recording period; common pipistrelle = 153 (130), soprano pipistrelle = 12 (17), Supplementary Table 1). Feeding buzzes were recorded within pipistrelle passes at all sites (min = 0.5 (1.0), max = 15.3 (12.0), Supplementary Table 1). And, overall pipistrelle activity (mean nightly pass totals recorded during inter-train gaps per 3.5 h recording period) ranged from min = 69.0 (33.3) to max = 356.0 (295.8) between sites.

Within the ≥ 90 s inter-train gaps, bat activity in the 30 s interval after a train had passed was approximately 50% of that recorded during the 30 s in-between trains, and the 30 s before a train arrived (for full details, see Fig. 1 and Table 1). But, activity in the 30 s following a train pass was unrelated to maximum SPL during the train pass, train noise frequency grouping (high or low), train length or wind speed (Table 2). Bat activity levels contrasted less between interval types as progressively longer intervals were compared. During the first 60 s and 120 s after a train pass (inter-train gaps ≥ 180 s; n = 1785, and ≥ 360 s; n = 1012, respectively), activity was around 30% lower than ‘before’ or ’between’ intervals of equal duration, with the contrast between ‘after’ and between intervals being only borderline significant. When comparing 240 s intervals (inter-train gaps ≥ 720 s; n = 462), activity during intervals between and after train passes was no longer significantly different, while activity intervals after train passes remained lower than during 240 s intervals before trains passed. For 480 s intervals (inter-train gaps ≥ 1440 s; n = 159) there were no significant differences between the interval types. Activity in the ‘before’ and ‘between’ intervals exhibited no significant differences at any interval duration.

**Table 1:** Parameter estimates (± 95% confidence intervals), z- and p-values for fixed effects included in linear models relating bat activity to interval type (before, after and between train passes), controlling for effects of nightly bat pass total (total bat passes recorded during inter-train gaps per 3.5 h recording period), time since start of recording, and mean windspeed/relative humidity during the recording period. Also, variance standard deviation and p-value of random effect used to account for repeated measures within locations for the 30 s interval model (see Methods).

Fixed effects	30 s intervals			60 s intervals			120 s intervals			240 s intervals			480 s intervals		
	Estimate	z	p	Estimate	z	p	Estimate	z	p	Estimate	z	p	Estimate	z	p
Interval category (before vs between)	0.15 ± 0.24	1.224	0.22	0.05 ± 0.25	0.43	0.67	0.05 ± 0.30	0.34	0.73	0.22 ± 0.37	1.20	0.23	0.07 ± 0.49	0.28	0.78
Interval category (after vs between)	− 0.63 ± 0.26	4.688	** < 0.0001**	− 0.34 ± 0.25	2.591	**0.01**	− 0.29 ± 0.30	1.888	0.06	− 0.26 ± 0.37	1.355	0.18	− 0.21 ± 0.48	0.83	0.41
Interval category (after vs before)	− 0.78 ± 0.26	5.80	** < 0.0001**	− 0.39 ± 0.25	3.00	**0.003**	− 0.34 ± 0.30	2.224	**0.03**	− 0.48 ± 0.38	2.51	**0.01**	− 0.27 ± 0.50	1.08	0.28
Nightly bat pass total	0.004 ± 0.001	9.305	** < 0.0001**	0.004 ± 0.001	10.54	** < 0.0001**	0.004 ± 0.001	4.00	** < 0.0001**	0.006 ± 0.002	7.68	** < 0.0001**	0.006 ± 0.003	4.38	** < 0.0001**
Time since start of recording	0.41 ± 0.12	6.517	** < 0.0001**	0.35 ± 0.12	5.615	** < 0.0001**	0.32 ± 0.16	3.95	** < 0.0001**	0.31 ± 0.22	2.834	**0.005**	0.16 ± 0.28	1.14	0.253
Mean windspeed	− 0.03 ± 0.04	1.047	0.30	− 0.01 ± 0.03	0.819	0.07	0.005 ± 0.04	0.228	0.82	− 0.02 ± 0.06	0.76	0.09	− 0.01 ± 0.09	0.09	0.927
Mean relative humidity	− 0.005 ± 0.01	0.60	0.55	− 0.01 ± 0.02	1.816	0.41	− 0.02 ± 0.02	1.715	0.09	− 0.02 ± 0.02	1.677	0.45	− 0.004 ± 0.01	0.93	0.35
**Random effect**	**Variance**	**SD**	**p**												
Location	0.04	0.2	0.06												

For the 30 s interval model, all parameters are averaged across the best fitting linear mixed-effect models (see “Methods”), except for the random effect variance, standard deviation and p-value which were extracted from the full model.

**Table 2:** Parameter estimates (± 95% confidence intervals), z- and p-values for fixed effects averaged across the best fitting linear models (see “Methods”) relating bat activity in the 30 s after a train pass with distance corrected maximum SPL during the train pass, train noise frequency group and train length, controlling for effects of nightly bat pass total (total bat passes recorded during inter-train gaps per 3.5 h recording period), time since start of recording, and wind speed.

Fixed effects	Estimate	z	p
Distance corrected maximum SPL	0.008 ± 0.22	0.16	0.88
Train noise frequency group (low)	0.03 ± 0.55	0.21	0.83
Train length	− 0.02 ± 0.20	0.22	0.83
Night total bat passes	0.58 ± 0.18	6.44	** < 0.0001**
Time since start of recording	0.49 ± 0.22	4.34	** < 0.0001**
Wind speed	− 0.03 ± 0.05	0.70	0.49

For all intervals analysed, bat activity was positively associated with the total number of bat passes per recording period, and time since start of recording (except for 480 s intervals, where time since start of recording was unrelated to activity).

## Discussion

Bat activity in the first 30 s after a train passed was around half that recorded during the 30 s intervals between and before train passes, neither of which differed significantly in activity level. Comparison of progressively longer intervals demonstrated this effect was relatively short-lived, and declined over time, with no significant difference in activity observed among eight-minute intervals recorded before, after and between passing trains. The interval between trains is likely to be the time when the influence of passing rail traffic is at its lowest, effectively providing an activity baseline. Therefore, these activity patterns demonstrate little or no effect of approaching trains. However, reduced activity after trains passed suggests a proportion of the bats present before a train arrived took temporary evasive action during, or shortly after the train pass. It remains possible that reduced sample sizes for longer intervals resulted in insufficient statistical power to detect longer term activity reductions. But, even the shortest-lived effect suggested by our analyses appears likely to yield negative consequences at common train densities, and especially so at busier sites. For example, a 120 s response would reduce activity for approximately one-fifth of the time available at sites with median rail traffic from our dataset (6 trains hr^-1^), rising to between one-half and two-thirds of the time available at the three busiest sites (15.6–19.3 trains hr^-1^). The activity changes we report imply repeated evasive action and/or exclusion from otherwise favourable environments, with the potential for commensurate opportunity or energetic costs. As a result, non-lethal disturbance effects of passing trains could disadvantage bats in most rail-side habitats. Accordingly, while moves to increase rail transport will benefit the global environment by reducing carbon emissions, such gains may come at the price of adverse effects on wildlife at a local level.

During straight flight, pipistrelles typically produce one echolocation pulse per wingbeat^48^. As such, reductions in activity recorded by static bat detectors should signify a corresponding decline in the numbers of bats present within range of the detector. Assuming reduced pipistrelle activity after train passes relates to a proportion of bats present moving to locations beyond detector range, then opportunity and/or energetic costs are likely to be incurred by these individuals. Linear landscape features are thought to attract pipistrelles by providing habitat suitable for three main functions; navigation, shelter from wind or predators, and foraging^28^, with the latter being detected through feeding buzzes at all sites we surveyed. Disruption of any of these critical activities could only be detrimental for exposed individuals. Additionally, while the comparative metabolic costs of different flight modes in pipistrelles have not been investigated, evasive action may be more energetically expensive than normal flight. Notably, data from *Rhinolophus* sp*.* indicate manoeuvring flight can be more metabolically demanding than horizontal flight, although this may depend on wing-loading^49^. Regardless, any impact would necessarily be proportional to the number of trains passing per unit time, which varied substantially between lines. Bats using the least busy branch line we investigated would only have experienced around one train every 30 min. Assuming the minimum 120 s effect suggested by our analyses, this situation translates to a roughly seven percent reduction in time available to exploit the rail-side habitat. But, on the busiest mainline, trains passed approximately every three minutes (on average) during our measurement periods. So, bats moving away from the area for two minutes each time a train passes would lose two-thirds of the time available for utilising such sites. It should be noted that the inevitable incremental reductions in our sample sizes as we analysed intervals of increasing duration may have reduced statistical power such that we underestimate the duration of reduced activity. Consequently, it is possible that activity is reduced for even greater proportions of available time, albeit probably at smaller overall percentages.

Such high levels of apparent disturbance would seem to imply that railways, and especially mainlines, provide poor bat habitat. Nevertheless, train density (site mean trains-per-hour) did not predict activity levels in any of our analyses. Also, the three sites with the highest pipistrelle activity were among the busier locations. And, all but two nights of our data were ‘high’ in common pipistrelle activity as classified by the Ecobat algorithm (Supplementary Table 1), which compares survey data with a reference dataset comprised of records from the same region, recorded at a comparable time of year^50^. Moreover, Ecobat assumes whole night recordings. As a result, its classification of our data will underestimate activity percentiles, as we only recorded for the first 3.5 h of each night. Plainly then, despite the passing trains, railway habitats appear favoured by pipistrelle bats, as has been reported elsewhere^22^. Yet, it is not clear whether this apparently strong association is as beneficial as high activity levels might suggest. Where linear landscape features may once have provided reliable cues of high-quality pipistrelle habitat, contemporary disturbance generated by rail traffic could have disrupted the historic relationship between cue and habitat quality. The railways we studied were all built between 1841 and 1884^51^, ostensibly providing sufficient time for novel habitat cue-quality relationships to be established. But, substantial variation in traffic since the lines were opened^52^, along with pollution and disturbance differences associated with the progression from steam, through diesel to electric locomotives is likely to have driven continuous change in habitat quality. Alternatively, given the substantial loss of habitat to intensive agriculture during the twentieth century^53^, it may be the case be that railway habitats are preferred not because they are optimal, but because better environments are no longer available.

Perhaps one of our most remarkable findings is the implication that around half the activity recorded as a train approached continued within the 20–25 m range of the detectors^49^ while the train passed. Furthermore, pipistrelle vocalisations remained visible in the spectrograms we used to identify bat calls during 80 out of the 1144 train passes we recorded (6.9%). As the noise created by passing trains mostly obscured all other sounds, this statistic is almost certainly an underestimate. It follows then, that an appreciable proportion of the pipistrelle populations using our sites remained in the vicinity of the railway, despite the apparent potential collision risk. Even so, continued activity does not preclude short range evasive action within the detectors’ range, and the reduced activity after train passes strongly suggests at least some bats were repelled to greater distances by oncoming trains. Both outcomes would be consistent with visual evidence that bats approaching roads reverse course when vehicles approach^30^, or when confronted with loud traffic noise^31^. Given the larger physical size and stimulus magnitude presented by passing trains, more pronounced responses might be expected in comparison to road vehicles. This does appear to be the case, in that Myczko et al.^54^ reported no variation in activity in relation to cars passing along Polish forest roads. Although Myczko et al.’s study assessed activity of a broader range of species, their lack of an observed effect may also be a consequence of relatively small sample size in comparison to our own.

The lack of relationship between activity after a train pass, and train sound intensity, speed or length is consistent with data demonstrating noise level, speed and vehicle type were not associated with decisions to change course when crossing roads^30^. But, our results contrast with a later study which found bats were more likely to turn to avoid vehicles when associated noise levels exceeded 88 dB^31^. Similarly despite evidence that higher bandwidth noise may be more aversive, and detrimental to foraging efficiency^34,35^, neither after-train bat activity, or site feeding buzz totals (*z* = 1.08, *p* < 0.28) were associated with train noise frequency grouping. These disparities may relate to species specific sensitivity differences, or greater stimulus variability among road vehicles when compared to trains. Equally, it may be the case that activity changes relating to train passes are primarily driven by other factors, such as light emitted, air turbulence, or simply the rapid movement of a large object. More generally, predictability may promote habituation^55^. As rail traffic moves along a fixed path, and is restricted to only a few relatively similar models (especially passenger trains), the stimuli they present could be less challenging to adapt to than those imposed by much less consistent road traffic.

Overall, our findings demonstrate that passing rail traffic is associated with reductions in bat activity, which may impose deleterious opportunity or energetic costs for individuals exploiting rail-side habitats. While, generally high common pipistrelle activity levels at all sites may indicate such costs are exceeded by advantages, it remains possible that rail-side habitats are acting as ecological traps. Many bat populations are thought to have suffered historic population declines, from which they are yet to recover^56^. This situation, along with the numerous contemporary pressures human activity places on bats^57^, demands action. Further investigations are now needed to better understand the consequences of global growth in rail transport for one of the largest^58^, yet most vulnerable and least well understood^59^ mammal groups.

## Methods

### Data collection

Data were collected between 15th July–20th September 2019 at 12 sites in south-eastern England (see Supplementary Table 1 for details), where woodland areas bordered a railway line, and access to the edge of the railway land was possible. Data gathering was contingent on weather conditions—minimum air temperature ≥ 10 °C, maximum wind speed ≤ 15 km/hr, and no precipitation. Where conditions diverged from these requirements on a given night, all data from that night (if any) was abandoned, and collection restarted the following night. All collected data (38 nights, 1–4 nights per site) were included in the analyses. Fieldwork protocols were approved by the University of Sussex Animal Welfare and Ethical Review Body (approval no. ARG/16/06).

### Bat activity

Pipistrelle activity was assessed by acoustic survey using static trackside bat detectors (SM2 + with omni-directional SMX-U1 ultrasonic microphone, Wildlife Acoustics, Maynard, US). Microphone sensitivity was tested for each detector prior to the start of data collection using the manufacturer’s ‘Ultrasonic Calibrator’ to ensure it met their minimum specification. Detectors were placed as close to the tracks as possible—usually adjacent to the railway boundary fence—with microphones mounted approximately 1.5 m above track level, perpendicular to the tracks, and pointing upwards at a 45º angle to horizontal. Distances between the microphone tip and the closest side of passing trains were recorded for each track using a laser measure (Tacklife HD40, Take Tools Co., Shenzen, China; precision ± 2 mm). Detector-train distances were 10.6 (3.2) m (mean (SD)), minimum = 5.2 m, maximum = 16.8 m, and train widths were 2.8 (0.1) m. Consequently, as the detectors have an approximate maximum 20–25 m range^49^, detected bats would be within 26.6–43.2 m of the track centre on the side facing the microphone, and within 1.8–18.4 m on the opposite side. Two detectors were deployed each night in case of malfunction, but only data from the one recording the most bat activity was used in the analyses. Bat detectors do not record false-positives, but there is some inter-detector variability. Therefore, data from the detector recording the most bat calls is the most accurate assessment of true activity.

Surveys took place from 30 min before sunset to three hours after sunset each night. Within this period, detectors were set to automatically trigger recording when the ultrasonic signal-to-noise ratio exceeded 18 dB. Sampling rate was set to 192 kHz, and an on-board digital 4 kHz high-band-pass filter applied (full settings are provided in Supplementary Materials Table 2, along with the settings file deployed on the detectors). Bat passes within the resulting sound files were defined as any call or series of calls separated by more than one second from another call or series of calls^60^. Individual bat passes were identified manually from spectrograms using Kaleidoscope v3.1.5 (Wildlife Acoustics, Maynard, US), with species, presence of feeding buzzes, and start/end time (to the nearest 100^th^ of a second) noted for each.

### Rail traffic activity

Rail traffic activity was evaluated from video footage of passing trains. Video recordings (1080p video, at 60 frames per second) were made continuously throughout each survey period using a small action camera (Action Cam L2, WiMiUS, Shenzen, China) mounted between the bat detector microphones. For each train appearing on the resulting footage, pass start and end time (the time points at which the train front or rear reached the centre of the field of view, respectively), track (either near or far), number of carriages and whether the pass overlapped with another were all recorded. Video and bat detector timings were calibrated using incidental sounds recorded on the video’s audio track, that were also picked up on the bat detector recordings.

Multiple models of train were operated along a number of the lines passing our survey sites, however it was not always possible to distinguish them after nightfall. Therefore, to estimate train length, we multiplied the mean car length of the five models used (21(1.47) m) by the number of cars (which was always discernible, regardless of lighting conditions). This estimate of train length was then used to calculate approximate train speed in km h^-1^ using the formula: (train length (m) / pass duration (s)) * 3.6.

### Train sound pressure levels and frequency spectra

The relative sound intensity of passing trains was determined from calibrated acoustic recordings. In addition to the ultrasonic microphone, each bat detector was equipped with an acoustic microphone (SMX-II, Wildlife Acoustics, Maynard, US) connected to the second channel. This channel was set to record continuously during the survey period (alongside the triggered ultrasonic channel), with a 1 kHz, 94 dB reference tone (Tenma Sound Level Calibrator 72–2680, Farnell, Leeds, UK) inserted at the start of each recording to allow subsequent calibration for sound pressure level (SPL).

SPL was extracted from the resulting audio files following the technique described by Švec & Granqvist^61^. Briefly, Praat v.6.1.09^62^ was used to generate an SPL curve for each acoustic audio file. Each curve was then calibrated by applying a multiplication factor derived from the difference between the automatically generated and known SPL value for the reference tone described above. The calibrated SPL curve was then exported using a minimum pitch of 5 Hz to provide approximately 6.25 readings per second (providing adequate temporal resolution while minimising dataset size). Maximum SPL for each train pass was defined as the highest value recorded between pass start and end times from the video data ± 5 s (as noise relating to track joins or curvature occasionally caused the highest SPL levels to occur slightly before/after the train front/rear was level with the video camera). To correct for differences in detector-train distance between sites, maximum SPL was standardised to maximum SPL at 1 m from the train using the sound attenuation equation SPL_2_ = SPL_1_ – 20*log(r_2_/r_1_), where SPL_2_ was maximum SPL at 1 m from the train, SPL_1_ was the measured maximum SPL, r_1_ was the detector-train distance in m, and r_2_ was 1 m.

Mean frequency spectra were also calculated (*meanspec* – R Package: Seewave 2.1.6^63^) for each train pass ± 5 s, and averaged (*mean_cl_boot* – R Package: Hmisc 4.5–0^64^) across each site (Supplementary Materials Fig. 1), and all train passes (Supplementary Materials Fig. 2). Visual inspection of site mean spectra revealed two distinct frequency groupings. The first group, comprising eight sites, was characterised by a gradual decline in energy between 5–20 kHz. Whereas, the second group—the four remaining sites (Cook’s Pond Viaduct, Fore Wood, Lewes Railway Land, Mill Wood), exhibited multiple distinct harmonic peaks above 5 kHz, as a result of ‘squealing’ and ‘flanging’ relating to track curvature and/or defects^32^. As within-site variation in train noise frequency spectra was limited, we included only frequency grouping in our statistical analyses (see below), rather than a train-pass-specific frequency measure.

### Weather conditions

Air temperature, relative humidity and wind speed during survey periods were sourced from Gatwick Airport Met Office weather station (the closest providing hourly weather data, situated 28.3(15.3) km from our survey sites; min/max weather station to survey site distance = 10.2/55.8 km)^65^. Mean values were calculated from all hourly measures made during a given survey period.

### Statistical analysis

All analyses were carried out using R v3.6.2^66^. Relationships between bat activity and passing trains were assessed by comparing numbers of bat passes recorded during intervals before, after and between train passes. Intervals during train passes (i.e. when any part of the train was level with the centre of the video camera’s field of view) were not analysed, as noise levels at these times were frequently of sufficient amplitude to obscure bat calls in detector spectrograms. Intervals before and after overlapping train passes (where, at two-track sites, two trains travelling in opposite directions were level with the video camera) were also excluded from the analyses. Such events likely present a markedly different stimulus to single trains, but were too rare (36 out of 1144 train passes overlapped) for statistical comparison.

Of 1088 inter-train gaps recorded (duration: mean (SD) = 391.5(568.4) s; median = 206 s; mode = 118 s), 856 were ≥ 90 s in duration. Evasive action lasting less than 30 s was assumed unlikely to materially influence time-budgets for bats using rail-side environments. Therefore, to balance including the most data possible with investigating the minimum period of evasive action likely to meaningfully affect bat behaviour, we initially compared bat activity between 30 s long intervals. Relationships between bat activity (response variable) and interval category (before, after or between train passes; explanatory variable) were assessed using generalized linear mixed models (*glmmTMB*—R Package: glmmTMB v1.0.1^67^). The same modelling approach was used to compare bat activity during after train intervals (response variable), and maximum SPL, train noise frequency grouping, train speed and train length (explanatory variables). A negative binomial distribution and log link function was selected for both models, with location specified as a random effect to account for repeated measures.

In addition to the effects of individual passing trains, we expected time of night and year, weather conditions, overall site activity and train density could influence the bat activity measured during each 30 s period. Activity varies over the course of both night and year^68,69^, potentially modulated by air temperature, humidity and wind^70^, and a positive correlation between activity assessed within a short period, and that night’s overall activity level is likely. Also, the overall amount of disturbance by trains at a site may influence habituation or sensitisation patterns^71^. Therefore, time since start of recording, date, mean air temperature, relative humidity and wind speed, nightly bat pass total (total bat passes recorded during inter-train gaps per 3.5 h recording period), inter-train gap duration, and train density (mean site trains-per-hour) were all considered as potential confounding explanatory variables. Potential confounding explanatory variables were included in the full multivariate models if they were associated with bat activity in preliminary univariate tests at *p* ≤ 0.1 (with the same random structure described above). Based on these preliminary analyses, only time since start of recording (bat activity: *z* = 6.01, *p* < 0.0001; bat activity in ‘after’ period: *z* = 4.24, *p* < 0.0001), wind speed (bat activity: *z* = 2.94; *p* < 0.003; bat activity in ‘after’ period: *z* = 2.43, *p* < 0.02), relative humidity (bat activity: *z* = 1.85; *p* < 0.06), and night total bat passes (bat activity: *z* = 6.90; *p* < 0.0001; bat activity in ‘after’ period: *z* = 6.82, *p* < 0.0001) were included in the full models.

To explore whether the association between bat activity and interval category lasted longer than 30 s, we ran four models with the same specification as those included in the averaged top performing 30 s period analyses (see below), doubling the interval duration over which bat activity was assessed each time (to 60 s, 120 s, 240 s and 480 s). This process reduced sample sizes incrementally (to 1785, 1012, 462 and 159 intervals respectively), as the minimum inter-train gap from which intervals could be sampled increased in duration (to 180 s, 360 s, 720 s and 1440 s).

Model selection was performed using an information theoretic approach, comparing AICc values between candidate models^72,73^. As we included only fixed effects likely to be influential on the basis of preliminary univariate tests, all possible combinations of fixed effects (without interactions) were ranked. Best performing model sets were determined using a threshold of ΔAICc ≤ 6 relative to the lowest AICc model^74^ (for model selection tables see Supplementary Materials Tables 3 and 4. Fixed effect parameter estimates were averaged across the best performing model set using *model.avg* (R package: MuMIn 1.43.17^75^). While in principle the location random effect should be incorporated into all models (to account for repeated measures), its effect in practice is negligible when among-location variance is very close to zero. Therefore, where random effect variance < 0.0001, we removed the random effect from the model to maintain parsimony^76,77^. Model assumptions were checked with *simulateResiduals* (R package: DHARMa v0.2.7^78^), *hat* (Base R) and check_collinearity (R package: performance v0.4.7^73^), and found to have been met. Model incidence ratio plots were created using *plot_model* (R package: sjPlot v 2.8.4^79^).

## Supplementary Information

Supplementary Information 1.

Supplementary Information 2.

## Data Availability

Raw data and all R-code are available to download from figshare, https://doi.org/10.25377/sussex.14787446.
