# Supplementary material for: Passing rail traffic reduces bat activity
Source: Sci Rep. 2021 Oct 19;11:20671. doi: 10.1038/s41598-021-00101-3 (PMC8526610; doi:10.1038/s41598-021-00101-3)
Supplement: Supplementary file 1 — Supplementary Information 1. [file 41598_2021_101_MOESM1_ESM.docx]

**Supplementary Table 1.** Site names, locations, survey dates, and summary statistics for all train and bat survey data analysed.

| **Site name** | | **Latitude** | **Longitude** | **Date** | **Train passes** | **Train passes per hour** | **Site mean train passes per hour** | **SD** | **Common pipistrelle** | |  | **Soprano pipistrelle** | |  | | | **Site mean pipistrelle passes per 3.5hr recording period** | | **SD** | | **Site mean pipistrelle feeding buzzes per 3.5hr recording period** | | **SD** |
| --- | --- | --- | --- | --- | --- | --- | --- | --- | --- | --- | --- | --- | --- | --- | --- | --- | --- | --- | --- | --- | --- | --- | --- |
|  |  |  |  |  |  |  |  |  | **Passes** | **Ecobat activity classification^1^** | **Feeding buzzes** | **Passes** | **Ecobat activity classification^2^** | **Feeding buzzes** | | |  |  |  |  |  |  |  |
| Hassocks | | 50.920643 | -0.148914 | 15/07/2019 | 55 | 15.7 | 15.6 | 0.2 | 54 | high | 2 | 4 | moderate | 0 | | | 104.3 | | 45.1 | | 8.3 | | 12.7 |
| Hassocks | | 50.920643 | -0.148914 | 16/07/2019 | 54 | 15.4 | 15.6 | 0.2 | 93 | high | 0 | 14 | moderate/high | 0 | | | 104.3 | | 45.1 | | 8.3 | | 12.7 |
| Hassocks | | 50.920643 | -0.148914 | 17/07/2019 | 55 | 15.7 | 15.6 | 0.2 | 146 | high | 23 | 2 | low/moderate | 0 | | | 104.3 | | 45.1 | | 8.3 | | 12.7 |
| Buxted | | 50.982898 | 0.127736 | 24/07/2019 | 8 | 2.3 | 1.7 | 0.8 | 197 | high | 16 | 3 | low/moderate | 0 | | | 207.0 | | 112.7 | | 15.3 | | 12.0 |
| Buxted | | 50.982898 | 0.127736 | 25/07/2019 | 3 | 0.9 | 1.7 | 0.8 | 316 | high | 27 | 7 | moderate | 0 | | | 207.0 | | 112.7 | | 15.3 | | 12.0 |
| Buxted | | 50.982898 | 0.127736 | 26/07/2019 | 7 | 2.0 | 1.7 | 0.8 | 91 | high | 3 | 7 | moderate | 0 | | | 207.0 | | 112.7 | | 15.3 | | 12.0 |
| Crowborough | | 51.051154 | 0.197991 | 31/07/2019 | 7 | 2.0 |  |  | 127 | high | 1 | 3 | low/moderate | 0 | | |  | |  | |  | |  |
| Sugworth Wood | | 51.023833 | -0.108444 | 05/08/2019 | 62 | 17.7 | 17.6 | 0.9 | 82 | high | 13 | 0 | low | 0 | | | 105.0 | | 45.6 | | 7.75 | | 7.3 |
| Sugworth Wood | | 51.023833 | -0.108444 | 06/08/2019 | 57 | 16.3 | 17.6 | 0.9 | 171 | high | 15 | 2 | low/moderate | 0 | | | 105.0 | | 45.6 | | 7.75 | | 7.3 |
| Sugworth Wood | | 51.023833 | -0.108444 | 07/08/2019 | 63 | 18.0 | 17.6 | 0.9 | 86 | high | 2 | 2 | low/moderate | 0 | | | 105.0 | | 45.6 | | 7.75 | | 7.3 |
| Sugworth Wood | | 51.023833 | -0.108444 | 08/08/2019 | 64 | 18.3 | 17.6 | 0.9 | 76 | high | 1 | 1 | low | 0 | | | 105.0 | | 45.6 | | 7.75 | | 7.3 |
| Petley Wood | | 50.932208 | 0.501421 | 12/08/2019 | 13 | 3.7 | 3.7 | 0.0 | 101 | high | 8 | 2 | low/moderate | 0 | | | 193.3 | | 78.7 | | 10.3 | | 2.1 |
| Petley Wood | | 50.932208 | 0.501421 | 13/08/2019 | 13 | 3.7 | 3.7 | 0.0 | 222 | high | 10 | 25 | moderate/high | 1 | | | 193.3 | | 78.7 | | 10.3 | | 2.1 |
| Petley Wood | | 50.932208 | 0.501421 | 15/08/2019 | 13 | 3.7 | 3.7 | 0.0 | 222 | high | 11 | 8 | moderate | 1 | | | 193.3 | | 78.7 | | 10.3 | | 2.1 |
| Valewood Lane | | 51.024053 | -0.402935 | 19/08/2019 | 21 | 6.0 | 6.5 | 0.4 | 117 | high | 2 | 2 | low/moderate | 0 | | | 209.7 | | 79.9 | | 2.3 | | 0.6 |
| Valewood Lane | | 51.024053 | -0.402935 | 20/08/2019 | 23 | 6.6 | 6.5 | 0.4 | 223 | high | 2 | 17 | moderate/high | 0 | | | 209.7 | | 79.9 | | 2.3 | | 0.6 |
| Valewood Lane | | 51.024053 | -0.402935 | 21/08/2019 | 24 | 6.9 | 6.5 | 0.4 | 260 | high | 3 | 10 | moderate/high | 0 | | | 209.7 | | 79.9 | | 2.3 | | 0.6 |
| Lewes Railway Land | | 50.870032 | 0.019331 | 22/08/2019 | 46 | 13.1 | 12.3 | 1.2 | 157 | high | 0 | 11 | moderate/high | 0 | | | 356.0 | | 295.8 | | 11.3 | | 12.9 |
| Lewes Railway Land | | 50.870032 | 0.019331 | 23/08/2019 | 45 | 12.9 | 12.3 | 1.2 | 638 | high | 25 | 59 | high | 0 | | | 356.0 | | 295.8 | | 11.3 | | 12.9 |
| Lewes Railway Land | | 50.870032 | 0.019331 | 24/08/2019 | 38 | 10.9 | 12.3 | 1.2 | 144 | high | 3 | 59 | high | 6 | | | 356.0 | | 295.8 | | 11.3 | | 12.9 |
| Mill Wood | | 51.160158 | -0.001459 | 26/08/2019 | 14 | 4.0 | 4.8 | 0.7 | 233 | high | 13 | 8 | moderate | 0 | | | 133.7 | | 97.1 | | 4.7 | | 7.2 |
| Mill Wood | | 51.160158 | -0.001459 | 27/08/2019 | 19 | 5.4 | 4.8 | 0.7 | 70 | high | 0 | 38 | high | 0 | | | 133.7 | | 97.1 | | 4.7 | | 7.2 |
| Mill Wood | | 51.160158 | -0.001459 | 28/08/2019 | 17 | 4.9 | 4.8 | 0.7 | 43 | high | 1 | 9 | moderate | 0 | | | 133.7 | | 97.1 | | 4.7 | | 7.2 |
| Wellgrove Wood | | 51.064420 | -0.143373 | 29/08/2019 | 72 | 20.6 | 19.3 | 1.5 | 43 | high | 3 | 2 | low/moderate | 0 | | | 69.0 | | 33.3 | | 5 | | 3.5 |
| Wellgrove Wood | | 51.064420 | -0.143373 | 30/08/2019 | 69 | 19.7 | 19.3 | 1.5 | 104 | high | 9 | 3 | low/moderate | 0 | | | 69.0 | | 33.3 | | 5 | | 3.5 |
| Wellgrove Wood | | 51.064420 | -0.143373 | 31/08/2019 | 62 | 17.7 | 19.3 | 1.5 | 50 | high | 3 | 5 | moderate | 0 | | | 69.0 | | 33.3 | | 5 | | 3.5 |
| Amberley Museum | | 50.893761 | -0.542017 | 01/09/2019 | 7 | 2.0 | 6.3 | 2.9 | 105 | high | 1 | 4 | moderate | 0 | | | 280.5 | | 188.2 | | 2.3 | | 1.0 |
| Amberley Museum | | 50.893761 | -0.542017 | 02/09/2019 | 27 | 7.7 | 6.3 | 2.9 | 543 | high | 3 | 6 | moderate | 0 | | | 280.5 | | 188.2 | | 2.3 | | 1.0 |
| Amberley Museum | | 50.893761 | -0.542017 | 03/09/2019 | 26 | 7.4 | 6.3 | 2.9 | 226 | high | 3 | 1 | low | 0 | | | 280.5 | | 188.2 | | 2.3 | | 1.0 |
| Amberley Museum | | 50.893761 | -0.542017 | 05/09/2019 | 28 | 8.0 | 6.3 | 2.9 | 237 | high | 2 | 0 | low | 0 | | | 280.5 | | 188.2 | | 2.3 | | 1.0 |
| Fore Wood | | 50.891817 | 0.491215 | 13/09/2019 | 16 | 4.6 | 3.7 | 0.6 | 59 | high | 0 | 47 | high | 2 | | | 76.5 | | 48.4 | | 0.5 | | 1.0 |
| Fore Wood | | 50.891817 | 0.491215 | 14/09/2019 | 12 | 3.4 | 3.7 | 0.6 | 67 | high | 0 | 56 | high | 0 | | | 76.5 | | 48.4 | | 0.5 | | 1.0 |
| Fore Wood | | 50.891817 | 0.491215 | 15/09/2019 | 11 | 3.1 | 3.7 | 0.6 | 28 | moderate/high | 0 | 34 | moderate/high | 0 | | | 76.5 | | 48.4 | | 0.5 | | 1.0 |
| Fore Wood | | 50.891817 | 0.491215 | 16/09/2019 | 13 | 3.7 | 3.7 | 0.6 | 11 | moderate/high | 0 | 4 | moderate | 0 | | | 76.5 | | 48.4 | | 0.5 | | 1.0 |
| Cook's Pond Viaduct | | 51.139735 | -0.004151 | 17/09/2019 | 20 | 5.7 | 5.7 | 0.2 | 144 | high | 7 | 1 | low | 0 | | | 114.5 | | 80.3 | | 2.5 | | 3.1 |
| Cook's Pond Viaduct | | 51.139735 | -0.004151 | 18/09/2019 | 21 | 6.0 | 5.7 | 0.2 | 45 | high | 2 | 0 | low | 0 | | | 114.5 | | 80.3 | | 2.5 | | 3.1 |
| Cook's Pond Viaduct | | 51.139735 | -0.004151 | 19/09/2019 | 19 | 5.4 | 5.7 | 0.2 | 54 | high | 0 | 0 | low | 0 | | | 114.5 | | 80.3 | | 2.5 | | 3.1 |
| Cook's Pond Viaduct | | 51.139735 | -0.004151 | 20/09/2019 | 20 | 5.7 | 5.7 | 0.2 | 213 | high | 1 | 1 | low | 0 | | | 114.5 | | 80.3 | | 2.5 | | 3.1 |
|  | 1. Reference range dataset included records only from within 30 days of the survey date, and within 100km^2^ of the survey location; n = 725 | | | | | | | | | | | | | |  |  | |  | |  | |  |  |
|  | 2. Reference range dataset included records only from within 30 days of the survey date, and within 100km^2^ of the survey location; n = 538 | | | | | | | | | | | | | |  |  | |  | |  | |  |  |

**Supplementary Table 2.** Settings applied to Wildlife Acoustics SM2+ bat detectors for recording simultaneous acoustic and ultrasonic audio.

| **Channel** | **Microphone** | **Frequency** |  |  |
| --- | --- | --- | --- | --- |
| Left | SMX-U1 | Ultrasonic |  |  |
| Right | SMX-II | Acoustic |  |  |
|  |  |  |  |  |
| **Hardware Settings** | **2.5V Bias** | **Gain** |  |  |
| Left | OFF | 12dB |  |  |
| Right | ON | 24dB |  |  |
|  |  |  |  |  |
| **Audio Settings** |  |  |  |  |
| Sample Rate | 192000 |  |  |  |
| Channels | Stereo |  |  |  |
| File Format | WAC0 | (WAC, no compression) | |  |
| Gain Left | 0.0 |  |  |  |
| Gain Right | 0.0 |  |  |  |
|  |  |  |  |  |
| **Advanced Audio Settings** |  |  |  |  |
| DIG HPF Left | 4000 | (fs/48) |  |  |
| DIG HPF Right | OFF |  |  |  |
| DIG LPF Left | OFF |  |  |  |
| DIG LPF Right | OFF |  |  |  |
| TRIG LVL Left | 18SNR |  |  |  |
| TRIG LVL Right | OFF |  |  |  |
| TRIG Max Length | 0 | (Meaning no limit on trigger length) | | |
| Bits | 16 | (Only of relevance to zero crossing) | | |
| Div Ratio | 16 | (Only of relevance to zero crossing) | | |
|  |  |  |  |  |
| **Schedule** |  |  |  |  |
| 01 AT SSET-00:30:00 |  |  |  |  |
| 02 DO |  |  |  |  |
| 03 RECORD 01:00:00 |  |  |  |  |
| 04 GOTO LINE 03 00X |  |  |  |  |
| 05 UNTSSET+03:00:00 |  |  |  |  |
| 06 GOTO LINE 01 00X |  |  |  |  |

**Supplementary Table 3.** Model selection table for a linear mixed-effect model relating bat activity to 30 s interval type (before, after and between train passes), controlling for effects of nightly bat pass total (total bat passes recorded during inter-train gaps per 3.5 hr recording period), time since start of recording and wind speed. All models included date, nested within survey site as a random effect term.

Global model: Bat pass rate ~ Interval category + Nightly bat pass total + Time since start of recording + Wind speed + Relative Humidity + (1 | Location)

**Supplementary Table 4.** Model selection table for a linear mixed-effect model relating bat activity in the 30 s after a train pass with distance corrected maximum SPL during the train pass, train length and train speed, controlling for effects of nightly bat pass total (total bat passes recorded during inter-train gaps per 3.5 hr recording period), time since start of recording, and wind speed. All models included date, nested within survey site as a random effect term.

Global model: Bat pass rate ~ Maximum SPL + Train noise frequency group + Train length + Nightly bat pass total + Time since start of recording + Wind speed + (1 | location)

**Supplementary Figure 1.** Mean train noise frequency spectrum of all train passes analysed. Shaded areas represent 95% confidence intervals.

**Supplementary Figure 2.** Mean train noise frequency spectra by survey site. Shaded areas represent 95% confidence intervals.
